# Influence of Chemically Disrupted Photosynthesis on Cyanobacterial Thylakoid Dynamics in *Synechocystis* sp. PCC 6803

**DOI:** 10.1038/s41598-019-42024-0

**Published:** 2019-04-05

**Authors:** Laura-Roxana Stingaciu, Hugh M. O’Neill, Michelle Liberton, Himadri B. Pakrasi, Volker S. Urban

**Affiliations:** 1 NScD, Oak Ridge National Laboratory, Oak Ridge, TN 37831 USA; 2 JCNS1, FZJ outstation at SNS, Oak Ridge National Laboratory, Oak Ridge, TN 37831 USA; 3 Department of Biology, Washington University, St. Louis, MO 63130 USA

## Abstract

The photosynthetic machinery of the cyanobacterium *Synechocystis* sp. PCC 6803 resides in flattened membrane sheets called thylakoids, situated in the peripheral part of the cellular cytoplasm. Under photosynthetic conditions these thylakoid membranes undergo various dynamical processes that could be coupled to their energetic functions. Using Neutron Spin Echo Spectroscopy (NSE), we have investigated the undulation dynamics of *Synechocystis* sp. PCC 6803 thylakoids under normal photosynthetic conditions and under chemical treatment with DCMU (3-(3,4-dichlorophenyl)-1,1-dimethylurea), an herbicide that disrupts photosynthetic electron transfer. Our measurements show that DCMU treatment has a similar effect as dark conditions, with differences in the undulation modes of the untreated cells compared to the chemically inhibited cells. We found that the disrupted membranes are 1.5-fold more rigid than the native membranes during the dark cycle, while in light they relax approximately 1.7-fold faster than native and they are 1.87-fold more flexible. The strength of the herbicide disruption effect is characterized further by the damping frequency of the relaxation mode and the decay rate of the local shape fluctuations. In the dark, local thicknesses and shape fluctuations relax twice as fast in native membranes, at 17% smaller mode amplitude, while in light the decay rate of local fluctuations is 1.2-fold faster in inhibited membranes than in native membranes, at 56% higher amplitude. The disrupted electron transfer chain and the decreased proton motive force within the lumenal space partially explain the variations observed in the mechanical properties of the *Synechocystis* membranes, and further support the hypothesis that the photosynthetic process is tied to thylakoid rigidity in this type of cyanobacterial cell.

## Introduction

In wild type *Synechocystis* sp. PCC 6803 (hereafter *Synechocystis* 6803) cyanobacterial cells the thylakoid membranes have a sheet-like conformation, forming an internal membrane system in the cytoplasm that follows the curvature of the cell envelope^1–3^. The structural organization of thylakoids in various types of cyanobacterial cells and in isolated membranes was thoroughly investigated by neutron diffraction in combination with other high-resolution structural methods^3–8^. It was found that, in general, photosynthetic membranes undergo massive structural changes as a response to changes in light conditions. However, the question of whether these structural changes also trigger dynamical changes and dynamical reorganization of the thylakoid membranes has not been answered until recently. In a high-resolution inelastic neutron scattering study on *Synechocystis* 6803, Stingaciu *et al*. (2016) showed for the first time the dynamical behavior of these respective photosynthetic membranes in living *Synechocystis* 6803 cells^9^. The dynamics was characterized in terms of bending elasticity using the theoretical approach for lipid bilayer membranes^10–15^. The undulation modes of the examined thylakoids were influenced by changes in the light conditions, suggesting a connection between the *Synechocystis* 6803 thylakoid membrane internal dynamics and the photosynthetic process^9^.

Photosynthesis is the process by which chlorophyll-containing organisms convert light energy into chemical energy through an electron transfer chain that is responsible for creating the proton motive force needed for ATP synthase^16^. DCMU (3-(3,4-dichlorophenyl)-1,1-dimethylurea) is an herbicide. When present in high concentrations DCMU disrupts the linear electron transport in photosynthetic membranes either by blocking the plastoquinone binding site^17–19^ or by inhibiting the oxidizing site of photosystem II^20^. These actions trigger disruption of the electron transfer chain and disruption of proton transport through the thylakoid lumen, affecting the reactions in non-cyclic photophosphorylation.

Our aim is to observe if there is a direct connection between the photosynthetic process and the undulation dynamics exhibited by living thylakoid membranes and to assess quantitatively the influence of inhibited photosynthesis on dynamics parameters. To examine the dynamics of the membranes in living cells we applied our previous Neutron Spin Echo approach^9^. Neutron Spin Echo (NSE) is a high-resolution spectroscopic technique that reveals dynamical information on time scale from few picoseconds to hundreds of nanoseconds and covers length scales from fractions of a nanometer to hundreds of nanometers. When combined with a structural study, the NSE technique provides accurate data on undulation modes that are too fast to be accessed by methods like photon correlation spectroscopy and on length scales that are too small to be resolved by dynamic light scattering. In order to observe the dynamic behavior of a particular structure one needs to tune the NSE experiment to observe “in real time” changes that happen only with respect to a particular structural organization (e.g. correlation times and specific correlation length scales), while contrast matching the rest of the larger structure. We used the structural data previously published by Liberton *et al*. (2013) which provided us with an architecture of the *Synechocystis* 6803 thylakoid membranes corresponding to repeated distances originating from single thylakoid membrane layer up to closely appressed membrane pairs^3^. We compared the dynamics of thylakoid membranes in the wild type *Synechocystis* 6803 cells, and in wild type *Synechocystis* 6803 cells inhibited by DCMU herbicide, during alternating light and dark cycles. The comparison is made in terms of bending fluctuations, thickness and shape fluctuations, and peristaltic and protrusion motions within the lumenal space using model theory for single bilayer membranes.

## Results and Discussion

We used the NSE spectrometer at the Spallation Neutron Source^21^ to obtain the intermediate scattering functions as relaxation data of both native and disrupted photosynthetic membranes. The intermediate scattering functions *S(q, t*)*/S(q*, 0*)* are the time-relaxation functions of correlated structures (distances) within cyanobacterial cells, characterized by the scattering vector *q*, which is inversely proportional to distance *d* = 2π/*q*, and relaxation time *t* inversely proportional to relaxation rate *Γ*, *t* = 1/*Γ*. The discussion of the data is organized as a comparison study between wild type native *Synechocystis* 6803 cyanobacterial cells (further named WT) and wild type *Synechocystis* 6803 cells inhibited by DCMU (further named WT-DCMU), under the same illumination conditions. For clarity, the figures of the manuscript are also presented as comparison panels between different samples and different illumination conditions. Throughout the following study the terms “*thylakoid membranes*” and “*cyanobacterial cells*” will refer to *Synechocystis* 6803 thylakoid membranes, while “*herbicide*” and “*photosynthetic inhibitor*” will refer to the DCMU herbicide.

### Membranes throughout the dark cycle

**Figure 1:**
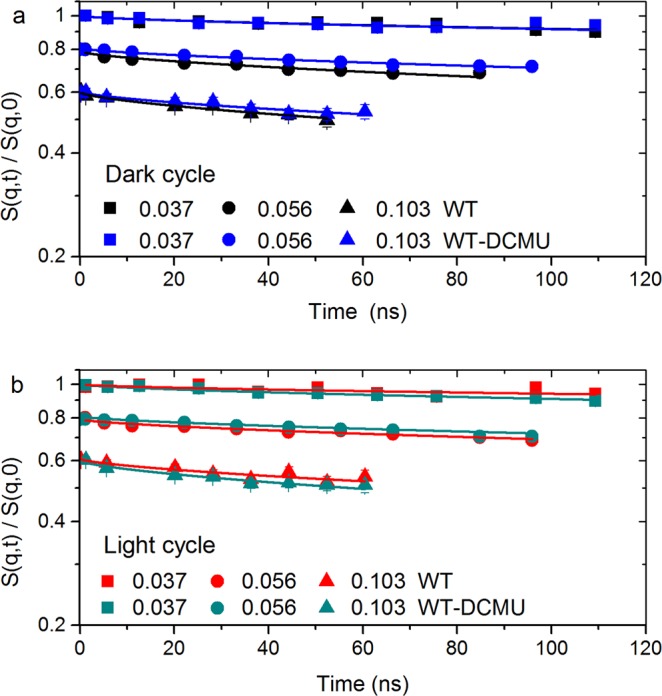
S(q, t)/S(q, 0) of native and inhibited cyanobacterial cells. All scattering functions start at unity and are shifted for better visualization. Solid lines represent fitting by a stretched exponential model^10,11^ according to Eq. (1); Only one *q* value per time-of-flight grouping for each sample is shown here (*note: the full set of data is available*). Panel “a” displays the evolution of membranes in dark and Panel “b” shows the evolution of membranes in light conditions. Error bars represent statistical error (1σ), in some cases smaller than the size of the symbol used.

The normalized intermediate scattering functions during the dark cycle for several selected scattering vectors *q* are shown in Fig. 1a. There is a clear trend in Fig. 1a showing that membranes in inhibited cells (WT-DCMU) relax slower than membranes in uninhibited cells (WT) in dark conditions. This difference in relaxation is considerable at some particular *q* values and it has been previously related to the bending rigidity of the photosynthetic membranes^9^. This suggests that native WT membranes are more flexible than the WT-DCMU membranes when both are in dark conditions.

To quantify this difference in rigidity, the intermediate scattering functions are fitted by stretched exponentials, with stretching exponent 2/3 as the Zilman-Granek model suggests for bilayer lipid membranes^10,11^ (see Methods). Table 1 contains the fitting result parameters of the selected *q*’s from Fig. 1a (dark cycle) with extended statistical information in Table S1 (Supplementary Information). The effective bending coefficients were calculated in the range where the Zilman-Granek theory is applicable and a linear *q*^3^ dependence of the relaxation rate is observed, e.g., *q* values higher than 0.07 Å^−1^ (d ≤ 90 Å). The effective bending coefficients are ~5655 *k*_B_*T* for the WT sample and ~8713 *k*_B_*T* for the WT-DCMU sample. These values are in good agreement with previously obtained bending coefficients of thylakoid membranes in dark and show indeed that during the dark cycle the inhibited membranes are 1.5-fold more rigid than the native membranes. Being more flexible, the WT membranes take less time to move through the available space, thus their faster relaxation of ~688 ns in the Zilman-Granek region as compared to ~1016 ns relaxation of inhibited membranes.

**Table 1:** Relaxation and bending properties with statistical errors (1*σ*) of native WT and inhibited WT-DCMU membranes during the dark cycle.

Sample	q (Å^−1^)	Γ × 10^−4^ (1/ns)	Γ/q^3^ (Å^3^/ns)	t (ns)	$\tilde{{\kappa }}$ (k_B_T)^**^
WT dark	0.037	2.58 ± 0.38	5.02 ± 0.85	3880.5 ± 564.8	—
WT dark	0.056	10.3 ± 0.83	5.79 ± 0.68	969.9 ± 78.2	—
WT dark	0.103	14.5 ± 0.96	1.29 ± 0.29	688.2 ± 45.4	—
WT dark at *y* intercept	—	—	1.08 ± 0.14	—	5654.85 ± 1420.5
WT-DCMU dark	0.037	2.68 ± 0.36	5.07 ± 0.82	3729.9 ± 491.7	—
WT-DCMU dark	0.056	4.26 ± 0.28	2.46 ± 0.41	2349.6 ± 156.9	—
WT-DCMU dark	0.103	9.83 ± 0.91	0.89 ± 0.29	1016.6 ± 94.3	—
WT-DCMU dark at *y* intercept	—	—	0.87 ± 0.06	—	8712.69 ± 1283.8

**Figure 2:**
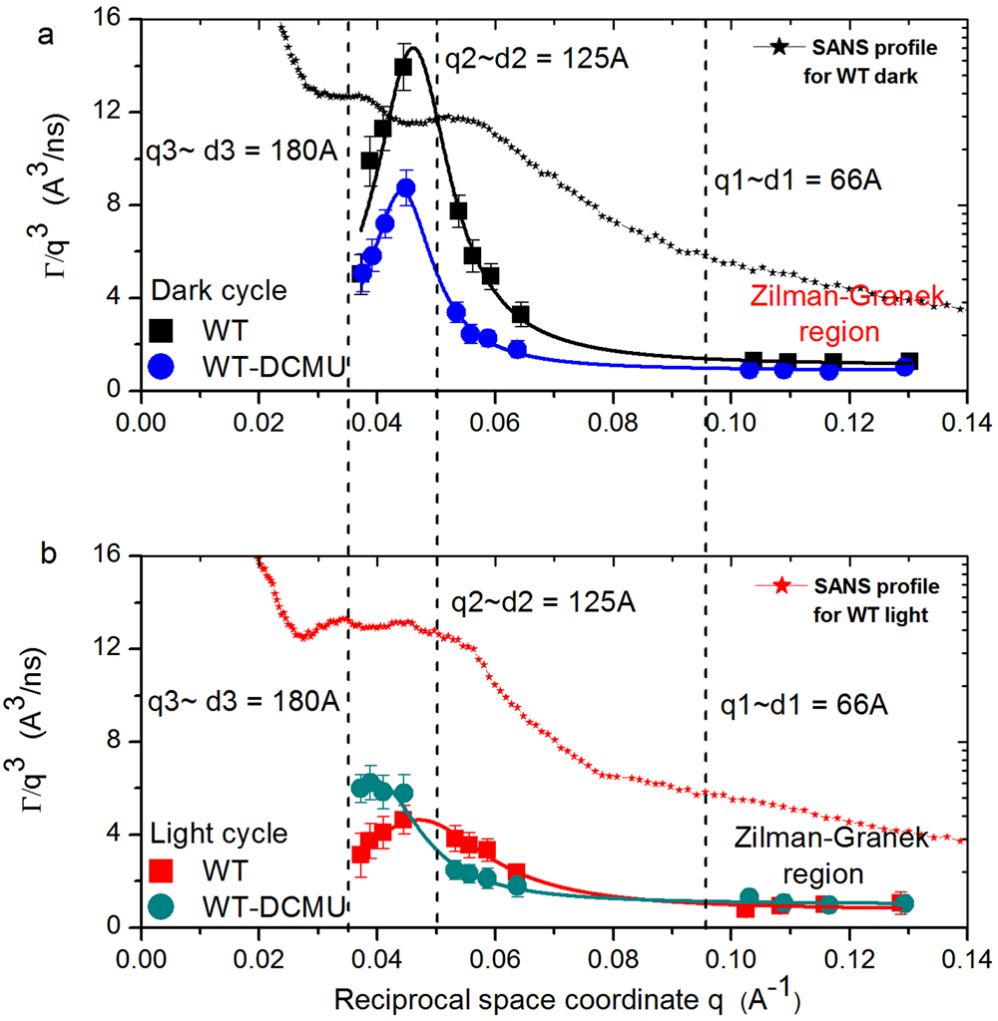
*q*^3^ dependence of the decay rate *Γ* for native and inhibited cyanobacterial cells. Panel “a” displays the membrane relaxation rates during the dark cycle and Panel “b” shows the relaxation rates during light conditions. Solid lines in the same color are the Lorentz fit according to Eq. (2). Distances are marked according to *q* probed by NSE, SANS profiles are superimposed, and the region where 1/*q* ≪ interthylakoidal distance is labeled Zilman-Granek region. Error bars represent statistical error (1σ), in some cases smaller than the size of the symbol used.

The relaxation parameters are calculated according to Eq. (1). The effective bending coefficient is calculated according to Eq. (3) from the *y* intercept of the Lorentz fit in Fig. 2a. **The value 0.00125 kg (m s)^−1^ is used as the D_2_O viscosity at 20 °C in the calculation of the bending modulus. Please see extended Table S1 (Supplementary Information) for comprehensive statistical errors.

Figure 2a shows the relaxation rate *Γ* for of all *q*’s measured. At high *q* values, the shallow linear *q*^3^ dependence of relaxation rate *Γ* represents the typical undulation motions in lipid bilayer membranes since length scales observed are shorter than distances between neighboring membranes^9–11^. Following the dependence of the decay rate *Γ* towards smaller *q* values, in Fig. 2a one can observe the strong deviation from *q*^3^ dependence with increasing correlation distances. This has been described as excess mobility to the underlying undulation dynamics, and a relation with illumination conditions was previously found. The same relation is observable here and will be discussed in detail later in the manuscript. To quantify the deviations from undulation dynamics (deviations from standard Zilman-Granek behavior), we used the approach established by Nagao and collaborators^12–15,22^ in which the excess motion is described as local shape fluctuations that include the peristaltic and protrusion motions of the membranes. Since there is a clear peak profile of the excess dynamics for both samples in dark conditions, a Lorentz function is used to describe the *q*^3^-normalized effective relaxation rate *Γ* as a sum of bending motion and local thickness fluctuation^13^ (see Methods). The solid lines in Fig. 2a are the fit results using the Lorentz function from Eq. (2). The fitting converged with a *χ*^2^ of 1.09 for WT sample and 0.19 for WT-DCMU. The best fit parameters are displayed in Tables 2 and S2 (Supporting Information), where *A* relates to the damping frequency of the peristaltic mode, *ξ* is the peristaltic mode amplitude, while the peak maximum *q*_0_ characterizes the local length scale at which the excess motions associated with local shape fluctuations are observed^12–15,22^. The product *A*·*q*_0_3 describes the decay rate of the local fluctuations. According to this, the local thickness and shape fluctuations relax twice as fast in native WT membranes as in DCMU inhibited membranes, at 17% smaller mode amplitude. For native WT membranes in dark, we obtain 1.34 × 10^6^ s^−1^, a value comparable to the characteristic relaxation rate of shape deformations of droplet emulsions^23^. This value decreases with the addition of DCMU to 6.65 × 10^5^ s^−1^ for WT-DCMU membranes in dark, indicating a stiffer behavior of the treated membranes, behavior comparable with the denser and compressed droplet emulsion from Gang *et al*.^23^. There is no observable deviation from the Lorentz function at higher *q* (distances smaller than the membrane thickness), where additional dynamics due to protrusion and diffusion of proteins at the membrane surface are usually sampled^13^. Therefore, one can safely assume that no protrusion or diffusion of proteins is observed within the NSE time window sampled here.

**Table 2:** Membranes shape fluctuation characteristics with statistical errors (1*σ*).

Sample	Γ_ZG_/q^3^ (Å^3^/ns)	q_0_ (Å^−1^)	A (Å^3^/ns)	ξ (Å)	A*q_0_^3^ × 10^−4^ (ns^−1^)	χ^2^
WT dark	1.08 ± 0.14	0.046	13.70 ± 1.2	130.8 ± 14	13.4 ± 1.63	1.09
WT-DCMU dark	0.87 ± 0.06	0.044	7.74 ± 0.3	153.8 ± 7.8	6.65 ± 0.35	0.19
WT light	0.73 ± 0.08	0.047	3.91 ± 0.2	69.9 ± 7.8	4.07 ± 0.37	0.15
WT-DCMU light	1.00 ± 0.07	0.039	5.29 ± 0.2	109.3 ± 10.4	3.34 ± 0.32	0.22

The parameters represent the Lorentz fit results of *q*^3^-normalized relaxation rate *Γ* using Eq. (2). *A* characterizes the damping frequency of the peristaltic mode, *ξ* is the peristaltic mode amplitude, *q*_0_ is the local length scale to observe thickness and shape fluctuations, and the product *A*·*q*_0_^3^ describes the decay rate of the local fluctuations. Please see extended Table S2 (Supplementary Information) for comprehensive statistical errors.

### Membranes throughout the light cycle

The normalized intermediate scattering functions for the same selected scattering vectors *q*, but during the light cycle, are shown in Fig. 1b. Here, the immediately visible trend is that inhibited WT-DCMU cells relax faster than native WT cells in light, opposite to what we observed during dark cycle. The intermediate scattering functions are again fitted by stretched exponentials, with the stretching exponent 2/3. The fitting results are presented in Tables 3 and S3 (Supporting Information). The effective bending coefficients calculated from the Lorentz fit intercept in Fig. 2b are ~12250 *k*_B_*T* for WT sample and ~6540 *k*_B_*T* for WT-DCMU sample, and the relaxation times in the Zilman-Granek region are ~1180 ns for WT sample and ~698 ns for WT-DCMU sample. The calculated parameters support the observations that during the light cycle the WT-DCMU inhibited thylakoid membranes relax more freely and take less time to move through the available space. Their relaxation is approximately 1.7-fold faster than native WT membranes and they are 1.87-fold more flexible. The spatial dependence of the decay rate *Γ* for both samples in light conditions in Fig. 2b shows excess mobility in addition to the underlying *q*^3^-dependent undulation dynamics as a peak profile. The peak widths and amplitudes are different than observed for dark conditions. The description of the peaks as local shape fluctuations (Eq. 2) yields the fit parameters displayed in Table 2, with a *χ*^2^ of 0.15 for WT sample and 0.22 for WT-DCMU sample in light. According to this the decay rate of the local fluctuations (*A*·*q*_0_^3^) is 1.2-fold faster in WT-DCMU inhibited membranes than in native WT membranes, at 56% higher amplitude. As under dark conditions, there is again no quantifiable dynamics due to protrusion or diffusion of proteins at distances smaller than the membrane thickness.

**Table 3:** Relaxation and bending properties with statistical errors (1*σ*) of native WT and inhibited WT-DCMU membranes during the light cycle.

Sample	q (Å^−1^)	Γ × 10^−4^ (1/ns)	Γ/q^3^ (Å^3^/ns)	t (ns)	$\tilde{{\kappa }}$ (k_B_T)^**^
WT light	0.037	1.59 ± 0.46	3.11 ± 0.94	6261.7 ± 1793.4	—
WT light	0.056	6.10 ± 0.48	3.54 ± 0.53	1638.8 ± 129.2	—
WT light	0.103	8.47 ± 0.11	0.79 ± 0.32	1180.6 ± 155.1	—
WT light at *y* intercept	—	—	0.73 ± 0.08	—	12250.10 ± 2707.3
WT-DCMU light	0.037	3.08 ± 0.19	5.99 ± 0.61	3249.9 ± 201.1	—
WT-DCMU light	0.056	3.98 ± 0.25	2.31 ± 0.38	2512.6 ± 160.4	—
WT-DCMU light	0.103	14.3 ± 0.99	1.31 ± 0.3	698.8 ± 48.2	—
WT-DCMU light at *y* intercept	—	—	1.00 ± 0.07	—	6540.62 ± 967.1

The relaxation parameters are calculated according to Eq. (1). The effective bending coefficient is calculated according to Eq. (3) from the *y* intercept of the Lorentz fit in Fig. 2a. **The value 0.00125 kg (m s)^−1^ is used as the D_2_O viscosity at 20 °C in the calculation of the bending modulus. Please see extended Table S3 (Supplementary Information) for comprehensive statistical errors.

### Comparison of the mechanical properties of native WT and inhibited WT-DCMU membranes

**Figure 3:**
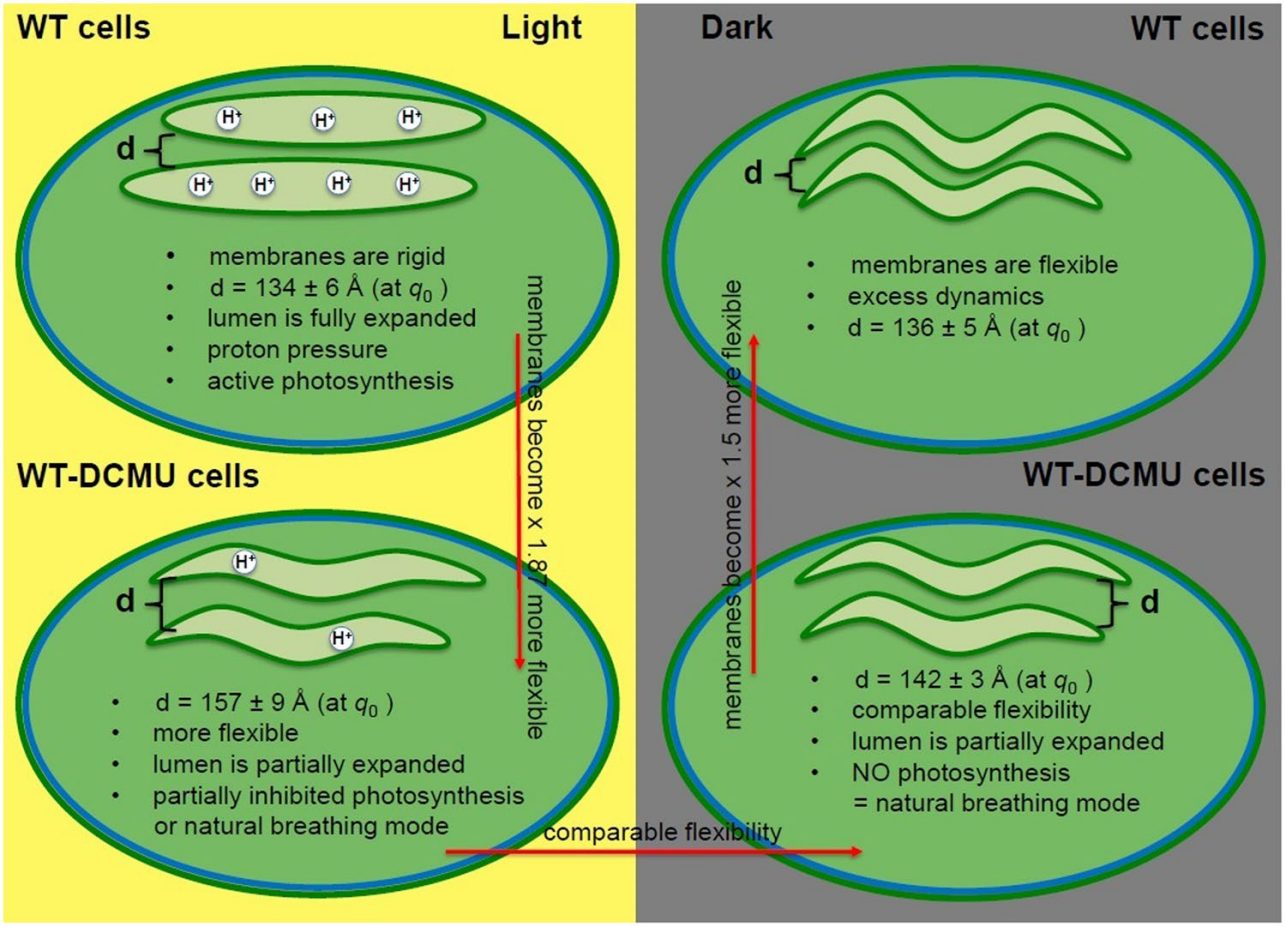
Summary diagram of the observed mechanical properties of native WT and inhibited WT-DCMU membranes. The green circles represent *Synechocystis* 6803 cyanobacterial cells with one pair of thylakoid membranes, under different illumination conditions. The distance *d* represents the interthylakoidal distance = the local length scale where thickness and shape fluctuations are sampled. The red arrows show the direction of increase flexibility.

The main phenomenon observed in our thylakoid membrane studies is, as previously noted^9^, the excess dynamics in addition to the expected *q*^3^-dependent bilayer undulation motion. This excess dynamic is represented by peak-like-shapes in addition to the linear dependence of *Γ* to *q*^3^, at different positions and of different amplitude and widths, corresponding to variations in sample composition and sample illumination conditions. From the detailed analysis of these peaks one can hypothesize about the complex dynamics processes that occur within the thylakoid membranes and their relationship with the photosynthetic process. These findings are summarized in the diagrams in Fig. 3.

For native WT membranes in D_2_O, the peak position *q*_0_ (Table 2) does not change considerably between the dark and light cycle. This translates to observing local motions over a distance ~1/*q*_0_ that does not change dramatically with time. Undulation motions are influenced by the apparent rigidity of the WT membranes. The rigidity of the membranes is related to their photosynthetic activity in a potentially quite complex way, considering the multiple steps and factors involved in the mechanisms of photosynthesis. The presence of a proton concentration gradient within the lumenal space during the light cycle creates pressurized membranes that appear stiffer and display restricted mobility (Fig. 3). Therefore, we have twofold higher apparent rigidity in light as compared to dark, and twofold smaller amplitude of the peristaltic mode. There is quite a difference in the damping frequency of the peristaltic mode and the decay rate of the local fluctuations centered at *q*_0_ between light and dark within the same WT membranes (Table 2). This relaxation mode may be visualized as local diffusivity that dissipates correlations at the length scale of membrane-pairs, which takes place faster in dark than in light. We suggest that the expansion of the thylakoid lumenal space due to build-up of proton pressure during photosynthesis leads to a more rigid structure and the observed slow-down of this relaxation mode, in agreement with previous findings^5,7,9^.

After adding DMCU to our samples, we still observe similar dynamic behavior, i.e. excess dynamics as a peak on top of base-line undulation motion, but with several significant differences. First, and most importantly, the excess dynamics of the membranes is not fully suppressed by DCMU, and peaks are still present in both light and dark cycles for the WT-DCMU membranes (Fig. 2). It was shown previously^3^ that the swelling of the thylakoid lumenal space as indicated by a diffraction maximum (labeled peak nr. 4 in Liberton *et al*. 2013) is suppressed by DCMU, in agreement with DCMU’s inhibition of PSII. A decrease in membrane mobility is still observed here by NSE for the DCMU treated cyanobacteria when comparing light with dark states. Therefore, the mobility of these thylakoids cannot be totally attributable to the lumenal swelling/de-swelling process due to build-up and loss of proton pressure alone. It could be an indication that the excess membrane dynamics, although visibly influenced by the presence of DCMU (when compared to native membranes), is a naturally occurring dynamics characteristic of free membranes within cytoplasm; a type of “*breathing*” relative motion of neighboring pairs relative to each other (Fig. 3); an innate flexibility and a dynamic component present at all times, and in all processes observed. During dark, this peristaltic mode of DCMU-inhibited membranes relaxes almost ~2-fold slower than in uninhibited native membranes (*A*, damping frequency of the peristaltic mode is 7.74 Å^3^/ns as compared to 13.70 Å^3^/ns) and with a higher mode amplitude *ξ*, at comparable local thickness, see Table 2. The entire dynamic process implies more rigid WT-DCMU membranes in dark as compared to more flexible native WT membranes in the dark, in agreement with their bending coefficients (8700 *k*_B_*T > *5600 *k*_B_*T*). During light, the DCMU peak function becomes higher than WT peak function (Fig. 2b) and shifts toward smaller *q* values. The excess dynamics of membranes suffers indeed from the disruption of the electron transport and proton pressure, in the manner we expected. Higher *Γ* values of the DCMU-WT membranes during light when photosynthesis should occur means faster relaxation and more flexible membranes. The effective bending coefficient of WT-DCMU membranes in light is ~6500 *k*_B_*T*, almost twofold smaller than the ~12250 *k*_B_*T* calculated for the WT membranes in light (Table 3). DCMU has inhibited PSII and disrupted the electron transfer chain, and the lack of proton pressure within the thylakoid lumen makes the inhibited membranes appear more flexible than the native ones. The strength of the DCMU disruption effect in light is also best characterized by the damping frequency *A* of the relaxation mode and the decay rate of the local shape fluctuations *A*·*q*_0_3 (Table 2). The damping frequency of DCMU inhibited membranes in light is higher than for native (5.29 Å^3^/ns > 3.91 Å^3^/ns), as expected for membranes that are more flexible. This value gets closer to the WT-DCMU membrane activity during dark, when photosynthesis effects should no longer influence the dynamics. The WT-DCMU membranes in light exhibit a higher relaxation mode amplitude (109.29 Å) and a stronger decay of the local shape fluctuations than the native WT membranes in light, in agreement with their flexibility.

The center of the peak has shifted toward smaller *q*_0_ (larger inter-thylakoidal distance) values for inhibited membranes, in both light and dark conditions, as compared to native ones, indicating that the observed correlated distance has increased (Fig. 3). It is conceivable that over time, after treatment with DCMU, in the absence of periodic swelling/de-swelling transitions due to buildup and alleviation of H+ pressure within lumenal space, the thylakoid membranes lose motility and arrange in stacks more tightly and appressed. This could appear as an effect similar to the dense and compressed droplet emulsions from Gang *et al*.^23^ and correspond to the transition between observing single thylakoid membrane layers and closely appressed membrane pairs^3,7^.

### Discussions on the photosynthetic behavior of inhibited WT-DCMU membranes

**Figure 4:**
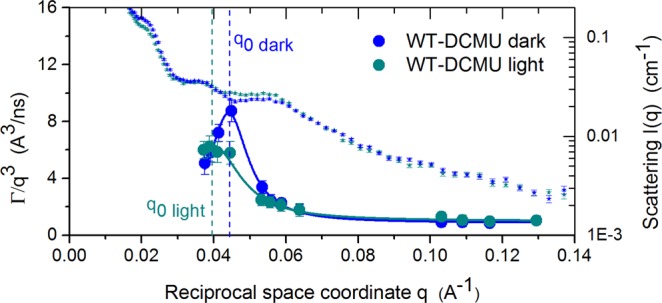
*q*-dependence of the *q*^3^-normalized decay rate *Γ/q*^3^ for DCMU inhibited cyanobacterial cells. A plot of membrane relaxation rate during dark and light conditions is superimposed by Small Angle Scattering data (small symbols). Solid lines in same color are the Lorentz fit according to Eq. (2). Peak position, *q*_0_, is marked, corresponding to length scale of probed local shape fluctuations. Error bars represent statistical error (1σ), in some cases smaller than the size of the symbol used.

Figure 4 shows the relaxation behavior of the WT-DCMU membranes superimposed by the structural data^3^. A difference is observed in the relaxation of the WT-DCMU membranes between light and dark cycle, even though DCMU should have suppressed those differences that otherwise arise from the influence of the photosynthetic process upon relaxation modes. Considering the multiple factors involved in the photosynthesis one can only speculate on the cause of the observed differences. First, and most obvious, will be that the amount of DCMU added to the sample and the exchange time involved were not enough for all the membrane stacks within the cyanobacterial cells to be fully inhibited. Therefore, the dynamics we observed during the light and dark cycle for the same WT-DCMU sample are still partially influenced by photosynthesis, and DCMU membranes are more flexible and exhibit relaxation motions with higher amplitude in the dark than in light. Second, due to the lack of proton motive force there is not enough ATP for the Calvin cycle, which may result in an increased pressure of NADPH on one side of the membrane and a non-releasing disrupted H+ pressure within the thylakoid lumen, so the inhibited membrane looks still a bit more rigid in light than in dark. Third, it was speculated that cyclic photophosphorylation, although not affected by DCMU, under certain circumstances can be enhanced by the presence of DCMU^24,25^. Therefore, even in the absence of non-cyclic electron transfer, cyclic photosynthesis maintains the thylakoid membranes as pressurized during light and they will appear slightly stiffer. Forth, it was shown recently that rapidly fluctuating light can produce field recombination-induced photo-damage (FRIP), where large spikes in electric field across the thylakoid membrane (*Δψ*) induce photosystem II recombination reactions that produce damaging singlet oxygen. FRIP is directly linked to the thylakoid proton motive force (pmf), and in particular, to the slow kinetics of partitioning pmf into *Δ*pH and *Δψ* components^26^. Variations in the local electric field due to the presence of DCMU could contribute to more rapid dissipation of *Δψ* component and formation of *Δ*pH.

Our observations align with the findings of Ünnep *et al*.^27^. Their data revealed a reversible smearing and broadening of the Bragg peak and an increased mosaicity of thylakoid membranes from chloroplasts, due to small variations in the pH of the interthylakoidal space. The effects of lowering the pH observed by Ünnep *et al*. (2017) were similar to those created by light-induced acidification of the lumen^27^, which in dynamics behavior translates as a reduction in the mobility. As stated before, the rigidity of the membranes relates to their photosynthetic activity in a very complex way if we consider the multiple steps and factors involved in the mechanisms of photosynthesis. Recently, Suga *et al*.^28^ showed in more detail that conformational changes due to change in illumination conditions occur at the oxygen-evolving complex site (OEC) in two different areas: around the QB/non-heme iron and at the Mn_4_CaO_5_ cluster. The conformational changes at the QB/non-heme iron position were assigned to the electron and proton transfer induced by illumination. The conformational changes at the Mn_4_CaO_5_ site suggest the occurrence of a new path for protons released from OEC^28^ during the transition from *S*_0_ to *S*_1_ state of the water-oxidation reaction process^29^. DCMU can act by either blocking the plastoquinone binding site (primary electron acceptor)^17–19^ or by inhibiting the oxidizing site (OEC) of photosystem II^20^. Insufficient DCMU concentrations or an affinity toward the primary electron acceptor terminal can leave a “freeway” for protons releasing within the thylakoid lumen, maintaining an active proton pressure and pressurized membranes that display restricted mobility.

Our results show that the membrane relaxation behavior is different between native WT cyanobacterial cells and DCMU inhibited cells. In the native cells the light/dark dynamics difference is comparable to what was observed previously^9^: the WT membranes stay more rigid in light due to electron transfer and proton motive force, and become very flexible when proton pressure is alleviated during the dark cycle. In the DCMU treated cells the difference between dark and light dynamics is smaller (~1.5 ratio of dark/light damping frequency) compared to the untreated cells (3.5 ratio of dark/light the damping frequency). This supports the hypothesis that photosynthesis plays a role in the dynamics and is affected by the presence of DCMU. The direct comparison between DCMU treated and untreated cells during the light cycle, when the effect of photosynthesis on the rigidity of the system is more observable, shows that by disrupting the electron transfer, DCMU affects the relaxation of the treated membranes as they become 1.87-fold more flexible than the untreated ones. Still, some inhibitory mechanical or chemical effects due to presence of DCMU exist. We do not fully understand at this point why the membranes of the DCMU treated cells appear less flexible compared to the native membranes during the dark cycle, when all electron transport process stops and one expects an identical behavior of the two types of membranes; or why the membranes of the same DCMU treated cells do not display the same identical flexibility in light as in dark. To the first, it is plausible that in DCMU treated cells the membranes of the thylakoid apparatus lose their general motility in time, and assume a stacked conformation that is more characteristic of non-living multilamellar stacks, which are known to be slightly more rigid structures. To the second, the dynamics accessed by NSE is a mixed process that on top of photosynthesis related dynamics contains an innate motility and natural dynamic component of the thylakoids, present at all times and unrelated to the photosynthetic process itself, and therefore only partially affected by the inhibitory presence of DCMU.

Overall, this study strengthens experimentally the hypothesis that the buildup in H+ pressure within lumenal space during the electron cycle drives important changes in the *Synechocystis* 6803 thylakoid membranes dynamics. Our data add a new dynamic dimension to the photosynthetic process in the presence of DCMU herbicides, indicating that photosynthesis can be directly and quantitatively correlated with the mechanical properties of photosynthetic membranes. The study points toward the flexibility of the thylakoids and their active function during energy conversion, rather than a rigid support frame for other photosynthetic components.

## Methods

### Sample preparation

*Synechocystis* sp. PCC 6803 was grown in Bg11 medium at 20 °C and 100 μE light intensity (100 μmol photons m^−2^ s^−1^) in one single batch. For native sample the cells were exchanged into D_2_O based Bg11 medium by sequential centrifugation and resuspension^3^ prior the neutron experiment. For DCMU treated sample the cells were processed in the same manner as for native and during the penultimate exchange in D_2_O based Bg11, the DCMU inhibitor (10 µM concentration) was added. The cells were incubated for 1 hour before centrifugation and final resuspension in 4 ml of growth media containing also 10 µM of DCMU.

### Inelastic Neutron Scattering

Neutron scattering experiments were performed on the NSE spectrometer at the Spallation Neutron Source^21^, Oak Ridge National Laboratory. As the sample holder, we used 4 mm-path length transparent quartz cells at 20 °C. A closed sample environment allowed alternating light and dark 12-hour cycles to mimic the circadian clock of the cells. The samples were adapted to different illumination conditions for 1 h before the neutron data collection started. The sample holder is equipped with a lighting apparatus that illuminates samples with cool white LED lights set at 100 μE in average intensity. We use incident wavelength of 11 Å accessing a dynamical range of 0.1 ns ≤ τmax ≤ 130 ns at momentum transfers corresponding to diffraction correlation peaks from the SANS diffraction patterns described in Liberton *et al*.^3^, which were measured at the Bio-SANS instrument^30^ of Oak Ridge National Laboratory. Solid angles of 0.035 Å^−1^ and 0.05 Å^−1^ and 0.095 Å^−1^ were measured, for both native WT and DCMU-WT samples in alternating 12 h light and dark conditions, in order to access repeated distances originating from single thylakoid membrane layer up to closely appressed membrane pairs in the range of 50 Å–175 Å^3,7^.

### Data analysis

NSE data were analyzed by grouping the wavelength spectra in four solid angles for better statistics on the *q* dependence. In order to characterize the membranes in terms of bending, peristaltic, and protrusion motions we implied the procedure already well established by Nagao *et al*. (2009) and Nagao *et al*. (2011)^13,14^ for investigating surfactant membranes, assuming the one-bilayer membrane model. The NSE data were fitted by a stretched exponential function with a stretching exponent of 2/3 suggested by Zilman & Granek^10,11^ for bilayer membranes:

1 $$\frac{S(q,t)}{S(q,0)}=\exp [-{({\rm{\Gamma }}t)}^{2/3}]$$

The Zilman-Granek model, based on mode coupling theory, explains the *q*^3^ dependence of the relaxation rate for systems where the hydrodynamic interactions dominate, and the wavelengths are shorter than the characteristic correlation lengths, in our case 1/*q* ≪ interthylakoidal distance. *Γ*/*q*^3^ represents in fact the linear dependence observed at *q* values higher than 0.07 Å^−1^ (*d* ≤ 90 Å). The later theory refined by Watson & Brown^31^ assumes a Lorentz function to express the deviations from the single membrane mode dynamics. The decay rate is, therefore, expressed as two additive terms: the first relates to bending fluctuations and the second one relates to thickness and shape fluctuation at the membrane scale^13^:

2 $$\frac{{\rm{\Gamma }}}{{q}^{3}}=\frac{{{\rm{\Gamma }}}_{ZG}}{{q}^{3}}+\frac{A}{1+{(q-{q}_{0})}^{2}\cdot {\xi }^{2}}$$

*Γ*_ZG_ is the decay rate due to bending fluctuation:

3 $${{\rm{\Gamma }}}_{ZG}=0.025\alpha \sqrt{\frac{{k}_{B}T}{\tilde{\kappa }}}\cdot \frac{{k}_{B}T}{{\eta }_{D2O}}\cdot {q}^{3}$$

with $\tilde{\kappa }$ = the effective bending modulus; *α* = scaling factor equal unity; *k*_B_*T* is the thermal energy; *η*_D2O_ is the viscosity of the solvent at T = 20 °C; *A* is the peak height of the Lorentzian connected to damping frequency of the peristaltic mode; *q*_0_ is the peak position and relates to the structure thickness determined by the form factor, and *ξ* is the peak width describing the mode amplitude. This assignment of the parameters in the Lorentz function comes from micro-emulsions theory^32^.

## Supplementary information

Supplementary Information
