# Supplementary material for: Influence of Chemically Disrupted Photosynthesis on Cyanobacterial Thylakoid Dynamics in Synechocystis sp. PCC 6803
Source: Sci Rep. 2019 Apr 5;9:5711. doi: 10.1038/s41598-019-42024-0 (PMC6450941; doi:10.1038/s41598-019-42024-0)
Supplement: Supplementary file 1 — Supplementary Information [file 41598_2019_42024_MOESM1_ESM.pdf]

Supplementary Information

Influence of Chemically Disrupted Photosynthesis on Cyanobacterial Thylakoid Dynamics in Synechocystis sp. PCC 6803

Authors: Laura-Roxana Stingaciu[1,2*], Hugh M. O'Neill[1], Michelle Liberton[3], Himadri B. Pakrasi[3], Volker S. Urban[1]

Affiliations:

[1]NScD, Oak Ridge National Laboratory, Oak Ridge, TN 37831, USA.

[2]JCNS1, FZJ outstation at SNS, Oak Ridge National Laboratory, Oak Ridge, TN 37831, USA.

[3]Department of Biology, Washington University, St. Louis, MO 63130, USA.

*Correspondence to: stingaciulr@ornl.gov

**Sample**	**q** (**Å^{-1}**)	**Γ** (**1/ns**)	**Γ** **err**	**χ^2**	**Γ/q^3** (**Å^3/ns**)	**Γ/q^3** **err**	**t** (**ns**)	**t** **err**	**$\tilde{\kappa}$** (**k$_B$T**) **	**$\tilde{\kappa}$** **err**
WT dark	0.037	2.58E-4	0.38E-4	0.91	5.02	0.85	3880.5	564.8	-	-
WT dark	0.056	10.3E-4	0.83E-4	0.42	5.79	0.68	969.9	78.2	-	-
WT dark	0.103	14.5E-4	0.96E-4	0.44	1.29	0.29	688.2	45.4	-	-
WT dark at *y* intercept	-	-	-	-	1.08	0.14	-	-	5654.85	1420.5
WT-DCMU dark	0.037	2.68E-4	0.36E-4	0.92	5.07	0.82	3729.9	491.7	-	-
WT-DCMU dark	0.056	4.26E-4	0.28E-4	1.15	2.46	0.41	2349.6	156.9	-	-
WT-DCMU dark	0.103	9.83E-4	0.91E-4	0.65	0.89	0.29	1016.6	94.3	-	-
WT-DCMU dark at *y* intercept	-	-	-	-	0.87	0.06	-	-	8712.69	1283.8

Table S1. Relaxation and bending properties with statistical errors (1σ) of native WT and inhibited WT-DCMU membranes during the dark cycle. The relaxation parameters are calculated according to equation (1) with χ^2 the goodness of Γ fit. The effective bending coefficient is calculated according to equation (3) from the *y* intercept of the Lorentz fit in Fig. 2a. ***The value 0.00125 kg (m s)$^{-1}$ is used as the D_2O viscosity at 20°C in calculation of the bending modulus*.

Sample	Γ_{ZG}/q^3 (Å^3/ns)	Γ_{ZG}/q^3 err	q_0 (Å^{-1})	q_0 err	A (Å^3/ns)	A err	ξ (Å)	ξ err	$A*q_0^3$ (ns^{-1})	$A*q_0^3$ err	χ^2
WT dark	1.08	0.14	0.0460	0.0005	13.70	1.2	130.8	14	13.4E-4	1.63E-4	1.09
WT-DCMU dark	0.87	0.06	0.0441	0.0002	7.74	0.3	153.8	7.8	6.65E-4	0.35E-4	0.19
WT light	0.73	0.08	0.0470	0.0006	3.91	0.2	69.9	7.8	4.07E-4	0.37E-4	0.15
WT-DCMU light	1.00	0.07	0.0398	0.0007	5.29	0.2	109.3	10.4	3.34E-4	0.32E-4	0.22

Table S2. Membranes shape fluctuation characteristics with statistical errors (1σ). The parameters represent the Lorentz fit results of the q^3-normalized relaxation rate Γ using equation (2), with χ^2 the goodness of fit. A characterizes the damping frequency of the peristaltic mode, ξ is the peristaltic mode amplitude, q_0 is the local length scale to observe thickness and shape fluctuations, and the product $A \cdot q_0^3$ describes the decay rate of the local fluctuations.

Sample	q ($Å^{-1}$)	Γ (1/ns)	Γ err	χ^2	Γ/q^3 ($Å^3$/ns)	Γ/q^3 err	t (ns)	t err	$\tilde{\kappa}$ (k_BT) **	$\tilde{\kappa}$ err
WT light	0.037	1.59E-4	0.46E-4	2.1	3.11	0.94	6261.7	1793.4	-	-
WT light	0.056	6.10E-4	0.48E-4	1.9	3.54	0.53	1638.8	129.2	-	-
WT light	0.103	8.47E-4	0.11E-4	1.09	0.79	0.32	1180.6	155.1	-	-
WT light at *y* intercept	-	-	-	-	0.73	0.08	-	-	12250.10	2707.3
WT-DCMU light	0.037	3.08E-4	0.19E-4	0.26	5.99	0.61	3249.9	201.1	-	-
WT-DCMU light	0.056	3.98E-4	0.25E-4	1.11	2.31	0.38	2512.6	160.4	-	-
WT-DCMU light	0.103	14.3E-4	0.99E-4	0.57	1.31	0.3	698.8	48.2	-	-
WT-DCMU light at *y* intercept	-	-	-	-	1.00	0.07	-	-	6540.62	967.1

Table S3. Relaxation and bending properties with statistical errors (1σ) of native WT and inhibited WT-DCMU membranes during the light cycle. The relaxation parameters are calculated according to equation (1) with χ^2 the goodness of Γ fit. The effective bending coefficient is calculated according to equation (3) from the *y* intercept of the Lorentz fit in Fig. 2a. ***The value 0.00125 kg (m s)$^{-1}$ is used as the D_2O viscosity at 20°C in calculation of the bending modulus*.
